# Clinical and Radiological Predictors for Early Hematoma Expansion After Spontaneous Intracerebral Hemorrhage: A Retrospective Study

**DOI:** 10.3390/neurolint17100170

**Published:** 2025-10-12

**Authors:** EJun Kim, Jee Hye Wee, Yi Hwa Choi, Hyuntaek Rim, In Bok Chang, Joon Ho Song, Yong Gil Hong, Ji Hee Kim

**Affiliations:** 1 Department of Neurosurgery, Hallym University College of Medicine, Anyang 14068, Republic of Korea; ejunkim@hallym.or.kr (E.K.); coldwings@naver.com (H.R.); nscib71@hanmail.net (I.B.C.); song@hallym.or.kr (J.H.S.); hongyk@hallym.or.kr (Y.G.H.); 2 Department of Otorhinolaryngology-Head and Neck Surgery, Hallym University College of Medicine, Anyang 14068, Republic of Korea; weejh07@gmail.com; 3 Department of Anesthesiology and Pain medicine, Hallym University College of Medicine, Anyang 14068, Republic of Korea; pcyhchoi@hallym.or.kr

**Keywords:** hematoma growth, hematoma expansion, imaging marker, intracerebral hemorrhage, predictor, progression

## Abstract

Background: Early hematoma expansion is a major determinant of poor outcomes after spontaneous intracerebral hemorrhage (ICH). Identifying reliable predictors of hematoma expansion may facilitate risk stratification and timely interventions. This study aimed to evaluate clinical, laboratory, and radiological factors associated with early hematoma expansion within 24 h. Methods: We retrospectively analyzed consecutive patients with spontaneous ICH admitted to a tertiary hospital in Korea between 2009 and 2021. Inclusion criteria were aged ≥ 18 years, primary spontaneous ICH, baseline non-contrast CT (NCCT), and follow-up CT within 24 h. Clinical, laboratory, and medication histories were collected, and NCCT/CT angiography (CTA) imaging markers (spot sign, blend sign, hypodensity, swirl sign, black hole sign, island sign, mean hematoma density) were evaluated. Early hematoma expansion was defined as an absolute volume increase ≥6 cm^3^ or a relative increase ≥33% on follow-up CT. Multivariate logistic regression identified independent predictors. Results: Among 899 screened patients, 581 met inclusion criteria (mean age 61.6 years; 59.7% male). Seventy-eight patients (13.4%) experienced early hematoma expansion. Independent predictors included CTA spot sign (adjusted OR 9.001, 95% CI 4.414–18.354), blend sign (OR 3.054, 95% CI 1.349–6.910), mean hematoma density <60 HU (OR 2.432, 95% CI 1.271–4.655), male sex (OR 2.902, 95% CI 1.419–5.935), and statin use (OR 2.990, 95% CI 1.149–7.782). Prior antiplatelet therapy was associated with a reduced risk of hematoma expansion (OR 0.118, 95% CI 0.014–0.981). Conclusions: Early hematoma expansion occurred in 13.4% of patients and was predicted by a combination of CTA and NCCT markers, as well as clinical and pharmacological factors. Spot sign remained the strongest predictor, while NCCT features such as blend sign and low hematoma density also provided practical prognostic value. These findings underscore the multifactorial pathophysiology of ICH expansion and highlight the importance of integrating imaging, clinical, and therapeutic variables into prediction models to improve early risk stratification and guide targeted interventions.

## 1. Introduction

Spontaneous or nontraumatic intracerebral hemorrhage (ICH) is a devastating subtype of stroke, defined as a bleeding into the brain parenchyma with or without intraventricular extension, and rarely, subarachnoid extension in the absence of trauma or surgery. ICH accounts for 10–20% of all strokes and is generally categorized as primary or secondary according to etiology [1]. Primary ICH, comprising 70–80% of cases, typically results from rupture of small vessels weakened by chronic hypertension or cerebral amyloid angiopathy [2], whereas secondary ICH arises from vascular malformations, coagulopathies, tumors, or medication use [3]. Despite advances in neurocritical care, the prognosis remains poor, with 30-day mortality exceeding 40% [4] and fewer than one-fifth of survivors regaining independent function at 6 months [5].

Early hematoma expansion—occurring in up to one third of patients within the first 24 h—is a key determinant of early neurological deterioration, mortality, and long-term disability [6,7]. Accordingly, substantial effort has focused on identifying predictors of hematoma expansion to enable early risk stratification and timely intervention. The CT angiography (CTA) “spot sign”, reflecting active contrast extravasation, is among the most validated radiological markers of ongoing bleeding [8,9]. However, CTA is not routinely available in many emergency settings and may be contraindicated in patients with renal dysfunction or contrast allergy; indeed, more than 80% of patients with ICH do not undergo CTA in clinical practice [10].

For these reasons, attention has shifted to non-contrast CT (NCCT) markers, which are widely available and practical. Several NCCT features—including blend sign, black hole sign, swirl sign, island sign, intrahematomal hypodensity, and overall hematoma heterogeneity—have been validated as significant predictors of hematoma expansion in both single-center and multicenter cohorts [11,12,13,14,15,16,17,18]. Meta-analyses further show that heterogeneity-related sign (swirl, blend, black hole, hypodensity) are consistently associated with increased risk of hematoma expansion and poor outcomes [15,18].

Beyond imaging, clinical and laboratory parameters—such as baseline hematoma size, blood pressure, leukocytosis, and prior antiplatelet therapy—have been proposed as modifiers of hematoma expansion risk [19,20]. Nonetheless, the relative contribution of these factors and their interaction with imaging markers remain incompletely defined, and results across studies are sometimes inconsistent.

Therefore, we conducted a comprehensive analysis integrating clinical, laboratory, and NCCT radiological variables to identify predictors of hematoma expansion within 24 h. Using our institutional cohort, we evaluated the impact of multiple risk factors—including NCCT heterogeneity signs and baseline clinical parameters—on early hematoma growth.

## 2. Methods

This study was approved by the Institutional Review Board of Hallym University (IRB No. HALLYM 2022-12-028). The requirement for written informed consent was waived due to the retrospective nature of the study.

### 2.1. Patient Selection

We conducted a retrospective observational study of consecutive patients age ≥ 18 years who were admitted with spontaneous ICH between April 2009 to January 2021. From an initial cohort of 899 patients, we excluded those with secondary ICH attributed to intracranial tumors, aneurysms, or other vascular malformations. Patients who underwent emergent hematoma evacuation before follow-up CT or who did not undergo repeat brain imaging within 24 h of the initial CT were also excluded.

### 2.2. Clinical Variables

Baseline demographic and clinical data were collected, including age, sex, time from symptom onset to ICH diagnosis, and Glasgow Coma Scale (GCS) at admission. Comorbidities included systemic hypertension, diabetes, liver disease, heart disease, chronic renal failure, familial history of ICH, prior cerebrovascular accidents (CVA), and cancer. Lifestyle factors assessed were alcohol consumption, smoking status, and body mass index (BMI). Laboratory data obtained at admission included white blood cell (WBC) count, platelet counts, activated partial thromboplastin time (aPTT), and international normalized ratio (INR). Systolic and diastolic blood pressure (SBP and DBP) were measured on admission. Medication history included prior use of antiplatelet or anticoagulant agents and lipid-lowering drugs.

### 2.3. Imaging Analysis

All NCCT scans were reviewed independently in a blinded fashion. Baseline hematoma volume was calculated using the ABC/2 technique. On the axial NCCT slice showing the largest hematoma area, the maximal diameter (A) and its longest perpendicular (B) were recorded. The approximate craniocaudal extent (C) was calculated as the number of CT slices containing hematoma multiplied by slice thickness. The estimated volume was then A × B × C/2, as previously validated [21]. Hematoma location was categorized as basal ganglia, thalamus, lobar, brainstem, cerebellum, others, or multiple sites. Additional features assessed included intraventricular hemorrhage (IVH), subarachnoid hemorrhage (SAH), white matter changes, and midline shift > 5 mm. The mean Hounsfield unit (HU) of the hematoma was also measured.

NCCT markers evaluated included island sign [22], margin irregularity [9], hypodensity [11], density heterogeneity [9], swirl sign [23], black hole sign [24], blend sign [13], and fluid level [25], defined according to published criteria (Supplementary Table S1). When available, CTA was reviewed for the presence of the spot sign.

### 2.4. Definition of Hematoma Expansion

Early hematoma expansion was defined as either a relative hematoma volume increase >33% or an absolute increase ≥6 cm^3^ on follow-up CT performed within 24 h of the baseline scan.

### 2.5. Statistical Analysis

Categorical variables were expressed as count (percentage), and continuous variables as mean ± standard deviation (SD) or median with interquartile range (IQR), as appropriate. Group comparisons were performed using the Chi-square test or Fisher’s exact test for categorical variables and Student’s t-test or Mann–Whitney U test for continuous variables. When nonparametric statistics are applied, we indicate them separately in the table. Odds ratios (ORs) with 95% confidence intervals (CIs) were calculated for discrete variables. Binary multivariate logistic regression was performed using the enter method, including all clinical, laboratory, and radiological variables collected in this study, regardless of univariate statistical significance. The following variables were entered: age (≥65), sex, symptom to CT time (<2.5 h) baseline hematoma volume (≥30 cm^3^), hematoma location (basal ganglia vs. others), mean hematoma density (<60 HU), CTA spot sign, island sign, margin irregularity, density heterogeneity, swirl sign, black hole sign, blend sign, fluid level, IVH, midline shifting, white matter change, SAH, CGS < 10, comorbid diseases, lifestyle factors, WBC ≥ 10,000, aPTT ≥ 40, INR ≥ 1.5, systolic and diastolic blood pressure, use of lipid lowering drugs, antiplatelet and anticoagulant therapy.

Two-tailed *p* values < 0.05 were considered statistically significant. Analyses were conducted using SPSS version 22.0 (IBM Corp., Armonk, NY, USA).

## 3. Results

Of 899 screened patients with spontaneous ICH, 112 were excluded due to secondary causes (tumor, aneurysm, vascular malformation), 56 underwent emergent hematoma evacuation before follow-up CT, and 150 lacked repeat imaging within 24 h. The final study population was 581. The mean age was 61.6 ± 13.8 years (range, 27–92 years), and 347 patients (59.7%) were male. The median time from symptom onset to baseline CT was 2.8 ± 4.1 (0.5–24). The mean baseline hematoma volume was 29.8 cm^3^ (range, 0.1–3857.8) The most common hematoma location was the basal ganglia (287 patients, 49.4%), followed by the thalamus (130, 22.4%), lobar regions (135, 23.2%), brainstem (43, 7.4%), and cerebellum (40, 6.9%); multiple or other sites accounted for the remainder. The mean hematoma density was 61.5 ± 6.2 HU (range, 34.8–80.4).

On baseline NCCT, hypodensities were present in 385 patients (66.3%), swirl sign in 185 (31.8%), island sign in 152 (26.2%), black hole sign in 78 (13.4%), blend sign in 73 (12.6%), and fluid level in 13 (2.2%). Among the 456 patients who underwent CTA, spot sign was observed in 70 (15.4%). IVH occurred in 221 patients (38.0%). The mean GCS at admission was 13 (mean 11.8 ± 3.6; range, 3–15). Comorbid conditions included systemic hypertension (50.6%), diabetes (17.0%), and prior CVAs (11.9%). Lifestyle factors included smoking (25.1%) and alcohol consumption (39.9%). Leukocytosis was present in 183 patients (31.5%), while 73 (12.6%) had aPTT ≥ 40 s and 31 (53%) had INR ≥ 1.5. Baseline medications included antiplatelet agents (6.5%), anticoagulants (4.6%), and lipid-lowering drugs (11.0%). Overall, 78 patients (13.4%) experienced early hematoma expansion within 24 h (Table 1).

Several factors were significantly associated with hematoma expansion (Table 2). Male patients had a higher expansion rate compared to females (15.9% vs. 9.8%, *p* = 0.037). Larger baseline hematoma volume (≥30 cm^3^) was associated with increased risk (15.0%, *p* = 0.013), as was shorter onset-to CT time (<2.5 h, *p* = 0.039). Basal ganglia location was more frequently associated with expansion compared to non-basal ganglia sites (17.4% vs. 9.5%, *p* = 0.005). Hematomas with a mean HU < 60 were strongly associated with expansion (21.5% vs. 8.6%, *p* < 0.001). Among imaging markers, the spot sign showed the strongest univariate association (40.0%, *p* < 0.001). Other significant predictors included hypodensity (17.1%, *p* < 0.001), density heterogeneity (homogenous density; categories 1 and 2; *p* < 0.001), swirl sign (18.9%, *p* = 0.008), black hole sign (23.1%, *p* = 0.007), and blend sign (23.3%, *p* = 0.008). Clinical and laboratory predictors included GCS < 10 at admission (18.2%, *p* = 0.036) and aPTT ≥ 40 s (26.0%, *p* <0.001). Other comorbidities and risk factors such as systemic hypertension, diabetes, smoking, alcohol use, or leukocytosis did not show significant associations.

Multivariate logistic regression identified six independent predictors of early hematoma expansion (Table 3). The CTA spot was the strongest risk factors, conferring an approximately nine-fold higher risk (adjusted OR = 9.00, 95% CI = 4.41–18.35, *p* < 0.001). Blend sign was also independently associated with expansion (adjusted OR = 3.05, 95% CI: 1.35–6.91, *p* = 0.007). Male sex conferred a nearly three-fold higher risk (adjusted OR: 2.90, 95% CI: 1.42–5.94, *p* = 0.004), while mean HU < 60 was associated with a more than two-fold increased risk (adjusted OR = 2.43, 95% CI = 1.27–4.66, *p* = 0.007). Lipid-lowering drug use was also associated with increased risk (adjusted OR = 2.99, 95% CI = 1.15–7.78, *p* = 0.025). In contrast, antiplatelet therapy was protective, significantly reducing the likelihood of expansion (adjusted OR = 0.12, 95% CI = 0.01–0.98, *p* = 0.048).

## 4. Discussion

In this large single-center cohort of patients with spontaneous ICH, early hematoma expansion occurred in 13.4% of cases. We identified six independent predictors: CTA spot sign, blend sign, mean hematoma density <60 HU, male sex, and statin use, whereas prior antiplatelet therapy appeared paradoxically protective. Unlike some previous studies that selected only variables significant in univariate analysis, we performed multivariate logistic regression using an enter method, including all available clinical, laboratory, and radiological predictors to ensure that potentially relevant factors were not omitted. These findings highlight the multifactorial nature of hematoma expansion and align with prior evidence emphasizing the role of both clinical and radiological factors in predicting hematoma growth.

The biological mechanisms underlying hematoma expansion remain incompletely understood but are essential to interpreting our results. Classically, ICH is initiated by rupture of a single small vessel, most often weakened by chronic hypertension or cerebral amyloid angiopathy [2,26]. Initial bleeding produces the core hematoma, and in some patients, clot stabilization and tissue counterpressure eventually limit further bleeding. The “avalanche model” suggests that the enlarging hematoma may mechanically disrupt adjacent fragile vessels, resulting in secondary ruptures and stepwise expansion [26,27]. Clinical imaging studies support this concept: irregular hematoma morphology, multiphasic bleeding patterns, and multiple foci of contrast extravasation on CTA have all been associated with ongoing bleeding [27]. Inflammatory pathways may also play an important role. Morotti et al. reported that elevated leukocyte and monocyte counts were associated with increased risk of hematoma expansion, whereas higher neutrophil counts paradoxically conferred a protective effect, suggesting a complex role of systemic inflammation [19]. This complements prior experimental work demonstrating that cytokines and endothelial dysfunction can increase vessel fragility and predispose to secondary bleeding.

Among imaging predictors, CTA spot sign was the strongest factor in our study (adjusted OR = 9.00, 95% CI = 4.41–18.35), consistent with prior multicenter analyses confirming its high predictive value [6,8,12]. A meta-analysis reported that the spot sign predicts hematoma expansion with an OR of 8.49 (95% CI: 7.28–9.90) and diagnostic performance of 62% sensitivity and 88% specificity [8]. Nevertheless, its clinical utility is limited by the fact that CTA is not routinely performed in acute settings, with more than 80% of ICH patients worldwide not receiving CTA [12]. Accordingly, NCCT markers have attracted increasing attention.

Our findings that blend sign (adjusted OR = 3.05, 95% CI = 1.35–6.91) and low mean density (<60 HU, adjusted OR = 2.43, 95% CI = 1.27–4.66) were independent predictors of hematoma expansion. These findings align with prior literature showing that hypodensity, heterogeneous density, blend sign, and black hole sign all predict hematoma expansion [9,11,12,13,14,16,17,18,23,24]. Boulouis et al. demonstrated the value of NCCT hypodensities [11], while Li et al. described novel NCCT signs such as the black hole and island signs as reliable predictors [22,24]. Previous studies have shown that blend sign has substantial correlation with spot sign (κ = 0.701) [13] and serves as a reliable predictor of poor outcome. A recent meta-analysis also confirmed that density heterogeneity-related signs are consistently associated with increased risk of expansion and poor outcome (OR = 3.49, 95% CI = 2.20–5.55) [18]. Recent literature has further shown that combining imaging markers with clinical data improves prediction of hematoma expansion. Huang et al. proposed a reliable grading system for basal ganglia ICH incorporating onset-to-CT time, baseline volume, and NCCT signs (island, blend) with excellent discrimination (AUC 0.918) [16]. Other investigators demonstrated that integrating intra-hematomal hypodensity with clinical BRAIN scores significantly increased predictive accuracy compared with either alone [17]. Our multivariate model, which integrates NCCT heterogeneity markers and key clinical variables such as sex and medication history, supports this integrated approach and provides real-world evidence for an Asian cohort. Additionally, several groups have developed composite scores combining NCCT features (blend, swirl, island) and baseline clinical parameters to standardize risk stratification [1]. Our data reinforce the concept that such combined models can improve beside prediction, especially where CTA is not routinely available.

In addition to imaging markers, we observed that male sex was independently associated with hematoma expansion, consistent with prior epidemiological data showing that men have different bleeding patterns and may be related to higher rates of alcohol consumption, smoking, and other vascular risk factors that could influence hemostatic function and vessel integrity [28].

The association between lipid-lowering medication use and increased hematoma expansion risk (adjusted OR = 2.99, 95% CI = 1.15–7.78) appears to contradict several studies that reported protective effects of statins in ICH patients [29]. However, our finding may reflect confounding by indication, as patients prescribed statins typically have multiple cardiovascular risk factors that could independently increase bleeding risk. Additionally, some studies have suggested that statin-associated benefits may be related to better overall medical management rather than direct neuroprotective effects. The relationship between statins and ICH outcomes remains controversial, with studies showing mixed results depending on study design, population characteristics, and adjustment for confounding variables.

Conversely, antiplatelet use was associated with reduced hematoma expansion risk in our study, which contradicts the established understanding that antiplatelet therapy increases bleeding risk and hematoma expansion in ICH patients [20]. However, this finding must be interpreted with extreme caution due to several important limitations. First, the sample size of antiplatelet users was very small (*n* = 38, 6.54% of total cohort), with only 2 patients (5.3%) experiencing hematoma expansion. Such small numbers are susceptible to statistical artifacts and may not reflect true associations. More importantly, selection bias and survivor bias likely influenced this result. Patients with severe ICH who were on antiplatelet therapy may have been more likely to die early or require emergent surgical intervention before follow-up imaging, thus being excluded from our analysis. Additionally, patients on antiplatelet therapy who survived to be included in the study may have represented a select group with milder hemorrhages or better baseline health status. The apparent protective effect could also reflect better overall medical management, as patients on antiplatelet therapy are typically under closer medical supervision and may receive more aggressive blood pressure control and supportive care.

Admission blood pressure was also assessed in our cohort. Although elevated systolic (SBP ≥ 180 mmHg) or diastolic (DBP ≥ 100 mmHg) pressures are recognized risk factors for hematoma growth [7,27], we did not observe significant associations (SBP ≥ 180 mmHg: *p* = 0.94; DBP ≥ 100 mmHg: *p* = 0.65). This may reflect early and standardized antihypertensive treatment at our center.

Our findings should also be interpreted in the context of prior cohort studies on hematoma expansion [11,30,31,32,33,34,35,36,37,38,39]. As summarized in recent large-scale investigation, multiple clinical and radiological factors have been validated as predictors of early hematoma expansion (Table 4). In addition to the above-mentioned factors, shorter time to baseline CT scan, hematoma volume, anticoagulants use or INR > 1.5, fluid-blood level, perihematomal edema, lower GCS, fibrinogen, deep hematoma location, IVH expansion, SBP, and irregular shape of hematoma were demonstrated.

## 5. Limitations

Several limitations of this study should be acknowledged. First, the retrospective and single-center design may limit the generalizability of our findings, and selection bias cannot be fully excluded. Second, follow-up imaging was restricted to within 24 h, which precludes the evaluation of delayed hematoma expansion and perihematomal edema dynamics that may also influence outcomes. Third, long-term functional outcomes were not assessed, making it difficult to determine the direct prognostic impact of the identified predictors on disability and mortality. Fourth, the number of patients with certain exposures, such as antiplatelet or statin therapy, was relatively small; thus, the observed associations—particularly the apparent protective effect of antiplatelets and the increased risk with statins—should be interpreted with caution and validated in larger, prospective cohorts. Fifth, although we collected multiple laboratory and radiological variables, residual confounding from unmeasured factors (e.g., hematoma growth rate before presentation, genetic predisposition, or detailed coagulation assays) cannot be excluded. Finally, imaging marker assessment was performed by radiologists with reference to established criteria, but inter-observer variability was not formally tested, which could affect reproducibility across institutions.

## 6. Conclusions

In this large single-center cohort, early hematoma expansion occurred in 13.4% of patients and was independently associated with CTA spot sign, low mean hematoma density, male sex, and statin use, while prior antiplatelet therapy showed a paradoxical protective effect. These results confirm the pivotal role of CTA and NCCT markers for risk stratification and highlight the need to integrate clinical and radiological factors into predictive models. Early recognition using accessible NCCT markers may be particularly valuable when CTA is unavailable, enabling timely interventions to reduce hematoma growth and improve outcomes.

## Figures and Tables

**Table 1:** Patient demographics and clinical characteristics.

Characteristics	Numbers (% of Cases or Range)
Total	581
Age (mean ± SD, years)	61.6 ± 13.8 (27–92)
Sex	
Male	347 (59.7%)
Female	234 (40.3%)
Symptom to CT (mean ± SD, hours)	2.8 ± 4.1 (0.5–24)
Hematoma volume (mean ± SD, cm^3^)	29.8 ± 161.4 (0.1–3857.8)
Hematoma location	
Basal ganglia	287 (49.4%)
Thalamus	130 (22.4%)
Brain stem	43 (7.4%)
Cerebellum	40 (6.9%)
Lobar	135 (23.2%)
Etc.	20 (3.4%)
Multiple	18 (3.1%)
HU (mean ± SD)	61.5 ± 6.2 (34.8–80.4)
Radiological markers	
Spot sign in the CT angiography ^†^	70 (15.4%)
Island sign	152 (26.2%)
Margin irregularity	
Category 1	161 (27.7%)
Category 2	167 (28.7%)
Category 3	127 (21.9%)
Category 4	52 (9.0%)
Category 5	74 (12.7%)
Hypodensities	
Type 1	182 (31.3%)
Type 2	180 (31.0%)
Type 3	10 (1.7%)
Type 4	13 (2.2%)
No hypodensity	196 (33.7%)
Density heterogeneity	
Category 1	186 (32.0%)
Category 2	176 (30.3%)
Category 3	113 (19.4%)
Category 4	54 (9.3%)
Category 5	46 (7.9%)
Swirl sign	185 (31.8%)
Black hole	78 (13.4%)
Blend sign	73 (12.6%)
Fluid level	13 (2.2%)
Preoperative CT findings	
IVH	221 (38.0%)
Midline shifting	97 (16.7%)
White matter change	153 (26.3%)
SAH	67 (11.5%)
GCS at admission (mean ± SD)	11.8 ± 3.6 (3–15)
Comorbid diseases or lifestyle factors	
Systemic hypertension	294 (50.6%)
Diabetes	99 (17.0%)
Liver disease	22 (3.8%)
Heart disease	27 (4.6%)
Chronic renal failure	10 (1.7%)
Smoking	146 (25.1%)
Alcohol consumption	232 (39.9%)
Familial history of ICH	123 (21.2%)
CVA history	69 (11.9%)
Cancer history	23 (4.0%)
Laboratory findings	
Leukocytosis (WBC ≥ 10,000)	183 (31.5%)
Thrombocytopenia (Platelet count < 100 × 10^9^/L)	30 (5.2%)
aPTT ≥ 40	73 (12.6%)
INR ≥ 1.5	31 (5.3%)
SBP ≥ 180	92 (15.8%)
DBP ≥ 100	120 (20.7%)
Medication	
Antiplatelet agents	38 (6.5%)
Anticoagulant agents	27 (4.6%)
Lipid lowering agents	64 (11.0%)
BMI (mean ± SD) ^‡^	23.8 ± 3.7 (13.3–41.2)
Early hematoma expansion	78 (13.4%) (13.3–41.2)

aPTT, activated partial thromboplastin time; BMI, body mass index; CVA, cerebrovascular accident; DBP, diastolic blood pressure; GCS, Glasgow coma scale; HU, Hounsfield unit; INR, international normalized ratio; ICH, intracerebral hemorrhage; IVH, intraventricular hemorrhage; SAH, subarachnoid hemorrhage; SBP, systolic blood pressure; SD, standard deviation; WBC, white blood cell. ^†^ 456 patients underwent CTA at admission. ^‡^ Missing data were included.

**Table 2:** Univariate analysis of predicting factors of the early hematoma expansion.

Characteristics	Total (*n* = 581)	Hematoma Expansion (*n* = 78)	No Expansion (*n* = 503)	*p* Value
Age ≥ 65	237	34 (14.3%)	203 (85.7%)	0.589
Sex				0.037 *
Male	347	55 (15.9%)	292 (84.1%)	
Female	234	23 (9.8%)	211 (90.2%)	
Symptom to CT time				0.039 *
Symptom to CT < 2.5 h	437	66 (15.1%)	371 (84.9%)	
Symptom to CT ≥ 2.5 h	144	12 (8.3%)	132 (91.7%)	
Hematoma ≥ 30 cm^3^	488	73 (15.0%)	415 (85.0%)	0.013 *
Hematoma location				0.005 *
Basal ganglia	287	50 (17.4%)	237 (82.6%)	
Non-basal ganglia	294	28 (9.5%)	266 (90.5%)	
Mean HU < 60	219	47 (21.5%)	172 (78.5%)	<0.001 *
Radiological markers				
Spot sign in CT angiography ^†^	70	28 (40.0%)	42 (60.0%)	<0.001 *
Island sign	152	23 (15.1%)	129 (84.9%)	0.473
Margin irregularity (Regular margin; categories 1, 2)	328	37 (11.3%)	291 (88.7%)	0.084
Hypodensity	385	66 (17.1%)	319 (82.9%)	<0.001 *
Density heterogeneity (Homogenous density; categories 1, 2)	362	35 (9.7%)	327 (90.3%)	<0.001 *
Swirl sign	185	35 (18.9%)	150 (81.1%)	0.008 *
Black hole sign	78	18 (23.1%)	60 (76.9%)	0.007 *
Blend sign	73	17 (23.3%)	56 (76.7%)	0.008 *
Fluid level	13	4 (30.8%)	9 (69.2%)	0.064
Preoperative CT findings				
IVH	221	35 (15.8%)	186 (84.2%)	0.182
Midline shifting	97	13 (13.4%)	84 (86.6%)	0.994
White matter change	153	17 (11.1%)	136 (88.9%)	0.328
SAH	67	9 (13.4%)	58 (86.6%)	0.998
GCS at admission < 10	165	30 (18.2%)	135 (81.8%)	0.036 *
Comorbid diseases or lifestyle factors				
Systemic hypertension	294	43 (14.6%)	251 (85.4%)	0.390
Diabetes	99	11 (11.1%)	88 (88.9%)	0.458
Liver disease	22	4 (18.2%)	18 (81.8%)	0.505
Heart disease	27	4 (14.8%)	23 (85.2%)	0.828
Chronic renal failure	10	1 (10.0%)	9 (90.0%)	0.749
Smoking	146	24 (16.4%)	122 (83.6%)	0.217
Alcohol consumption	232	36 (15.5%)	196 (84.5%)	0.228
Familial history of ICH	123	20 (16.3%)	103 (83.7%)	0.299
CVA history	61	7 (11.5%)	54 (88.5%)	0.637
Cancer history	23	2 (8.7%)	21 (91.3%)	0.497
Laboratory findings				
Leukocytosis (WBC ≥ 10,000)	183	21 (11.5%)	162 (88.5%)	0.350
Thrombocytopenia (Platelet count < 100 × 10^9^/L)	30	5 (16.7%)	25 (83.3%)	0.593
aPTT ≥ 40	73	19 (26.0%)	54 (74.0%)	<0.001 *
INR ≥ 1.5	30	7 (23.3%)	23 (76.7%)	0.103
SBP ≥ 180	91	12 (13.2%)	79 (86.8%)	0.942
DBP ≥ 100	123	15 (12.2%)	108 (87.8%)	0.652
Medication				
Antiplatelet agents	38	2 (5.3%)	36 (94.7%)	0.127
Anticoagulant agents	27	5 (18.5%)	22 (81.5%)	0.411
Lipid lowering agents	64	10 (15.6%)	54 (84.4%)	0.584
BMI ≥ 30 ^‡^	32	6 (18.8%)	26 (81.3%)	0.366

aPTT, activated partial thromboplastin time; BMI, body mass index; CVA, cerebrovascular accident; DBP, diastolic blood pressure; GCS, Glasgow coma scale; HU, Hounsfield unit; INR, international normalized ratio; ICH, intracerebral hemorrhage; IVH, intraventricular hemorrhage; SAH, subarachnoid hemorrhage; SBP, systolic blood pressure; SD, standard deviation; WBC, white blood cell. ^†^ 456 patients received CTA at admission. ^‡^ Missing data were included. * Significance < 0.05.

**Table 3:** Binary multivariate logistic analysis to predict factors for the early hematoma expansion.

Factors	Adjusted Odds Ratio	95% Confidence Interval	*p* Value
Spot sign	9.00	4.41–18.35	<0.001
Blend sign	3.05	1.35–6.91	0.007
Male	2.90	1.42–5.94	0.004
Mean HU < 60	2.43	1.27–4.66	0.007
Lipid lowering agents	2.99	1.15–7.78	0.025
Antiplatelet agent	0.12	0.01–0.98	0.048

HU, Hounsfield unit.

**Table 4:** Recent cohort studies reporting outcomes and predictors of hematoma expansion in spontaneous intracerebral hemorrhage.

Year/Authors	Country	No. of Patients	No. of Hematoma Expansion (% of Total Patients)	Follow-Up CT Time from Onset (Hours)	CT Predictor of Hematoma Expansion	Clinical Predictor of Hematoma Expansion
Boulouis et al./2016 [11]	USA	1029	224	Within 48 h from symptom onset	NCCT hypodensity, CTA spot sign, irregular shape, blend sign	Shorter time to CT (<6 h), warfarin use
Morotti et al./2016 [19]	USA	1302	207	Not reported		Leukocyte count
Li et al./2017 [22]	China	252	85	Baseline ≤ 6 h	Island sign	
Morotti et al./2017 [12]	USA et al.	989	186 (21.4%)	Baseline CT ≤ 4.5 h, follow-up CT 24 h	Hypodensities, blend sign, irregular hematoma shape, heterogeneous density	
Huang et al./2018 [16]	China	266	99 (37.22%)	Within 24 h	Blend sign, island sign, swirl sign, IVH	Time to baseline CT scan, baseline hematoma volume, anticoagulants use or INR > 1.5
Miyahara et al./2018 [37]	Japan	622	10.8%	Not reported	HEAVN score: Heterogeneity, Niveau (fluid–blood level), Edema, Volume > 30 mL, Anticoagulant	
Sakuta et al./2018 [38]	China	382	6 h: 57/380 24 h: 44/381	6 h, 24 h, 7 days	NAG score: Niveau (fluid–blood level), Anticoagulant, Globular density	
Morotti et al./2018 [40]	Italy et al.	1539	Not reported	Within 24–48 h	BAT score = Blend sign (1) + Any hypodensity (2) + Time < 2.5 h	
Liu et al./2019 [41]	China	1157	246	≤72 h	SVM mode: male, time to initial CT scan, GCS, fibrinogen, black hole sign, blend sign	
Roh et al./2020 [36]	USA	1457	Not reported	Within 2 days	Deep hematoma location	
Yogendrakumar et al./2020 [35]	Canada et al.	256	80	24 h	IVH expansion	
Murthy et al./2021 [20]	USA et al.	1420	279 (19.6%)	72 h		Prior antiplatelet therapy (not associated with hematoma expansion)
Chen et al./2022 [39]	China	223	27 (12.1%)	Within 48–72 h	Perihematomal edema expansion	Age, female sex, lower GCS, higher SBP, larger hematoma volume
Morotti et al./2024 [34]	Italy et al.	1472	223 (15.2%)	Within 24–72 h	NCCT hypodensities, blend sign, heterogeneous density, irregular shape, CTA spot sign	Age, anticoagulants use, lower GCS, shorter time from symptom to CT, larger hematoma volume, ultra-early hematoma growth
Ironside N et al./2024 [30]	USA et al.	340	112 (32.9%)	24 ± 6 h	Larger hematoma size, density heterogeneity, shape irregularity, peripheral density distribution	
Present study	South Korea	581	78 (13.4%)	Within 24 h	Blend sign, mean HU < 60	Male sex, lipid-lowering drugs

CTA, CT angiography; GCS, Glasgow coma scale; HU, Hounsfield unit; INR, international normalized ratio; IVH, intraventricular hemorrhage; NCCT, non-contrast CT; SBP, systolic blood pressure.

## Data Availability

The raw data supporting the conclusions of this article will be made available by the authors on request.

## Supplementary Materials

The following supporting information can be downloaded at: https://www.mdpi.com/article/10.3390/neurolint17100170/s1, Table S1: Radiological markers according to the previously published criteria.
