# Supplementary material for: Clinical and Radiological Predictors for Early Hematoma Expansion After Spontaneous Intracerebral Hemorrhage: A Retrospective Study
Source: Neurol Int. 2025 Oct 12;17(10):170. doi: 10.3390/neurolint17100170 (PMC12566760; doi:10.3390/neurolint17100170)
Supplement: Supplementary file 1 [file neurolint-17-00170-s001.zip › neurolint-3883845-supplementary.pdf]

Supplementary table S1: Radiological markers according to the previously published criteria.

	Description or characteristics
Island sign	(1) ≥3 scattered small hematomas all separate from the main hematoma or<br>(2) ≥4 small hematomas some or all of which may connect with the main hematomas on 1 axial slice of hematoma
Margin irregularity	5-point categorical scales were created: 2 scales ranged from Category I (most regular shape) to Category 5 (most irregular shape)
Hypodensities	4 types of hypodensities were defined based on the distinctness of their margins and their relative density<br>Type 1: a brain-like density and distinct margins<br>Type 2: a brain-like density and indistinct margins<br>Type 3: an edematous or cerebrospinal fluid-like density<br>Type 4: a mixed density with a fluid-fluid level
Density heterogeneity	5-point categorical scales were created: 2 scales ranged from Category I (most homogeneous density) to Category 5 (most heterogeneous density)
Swirl sign	A hypo- or isodensity within a region of a hyperdensity
Black hole sign	(1) Relatively hypoattenuated area (Black hole) encapsulated within the hyperattenuating hematoma<br>(2) The black hole could be round, oval, or rod-like but was not connected with the adjacent brain tissue<br>(3) The relatively hypoattenuated area should have an identifiable border<br>(4) The hematoma should have at least a 28 Hounsfield unite difference between the 2 density regions
Blend sign	(1) Blending of relatively hypoattenuating area with adjacent hyperattenuating region within a hematoma

	(2) There is a well-defined margin between the hypoattenuating area and adjacent hyperattenuating region that is easily recognized by the naked eye<br>(3) The hematoma should have at least an 18 Hounsfield unit difference between the 2 density regions<br>(4) The relatively hypoattenuating area was not encapsulated by the hyperattenuating region
Fluid level	As a horizontal interface between hypodense bloody serum layered above hyperdense settled blood
